# Change in energy expenditure and physical activity in response to aerobic and resistance exercise programs

**DOI:** 10.1186/s40064-015-1594-2

**Published:** 2015-12-22

**Authors:** Clemens Drenowatz, George L. Grieve, Madison M. DeMello

**Affiliations:** 1 Department of Exercise Science, University of South Carolina, Columbia, SC 29208 USA; 2 Arnold School of Public Health, Department of Exercise Science, University of South Carolina, 921 Assembly Str., Public Health Research Center, Columbia, SC 29208 USA

**Keywords:** Aerobic training, Resistance training, Total daily energy expenditure, Moderate-to-vigorous physical activity, Energy balance

## Abstract

Exercise is considered an important component of a healthy lifestyle but there remains controversy on effects of exercise on non-exercise physical activity (PA). The present study examined the prospective association of aerobic and resistance exercise with total daily energy expenditure and PA in previously sedentary, young men. Nine men (27.0 ± 3.3 years) completed two 16-week exercise programs (3 exercise sessions per week) of aerobic and resistance exercise separated by a minimum of 6 weeks in random order. Energy expenditure and PA were measured with the SenseWear Mini Armband prior to each intervention as well as during week 1, week 8 and week 16 of the aerobic and resistance exercise program. Body composition was measured via dual x-ray absorptiometry. Body composition did not change in response to either exercise intervention. Total daily energy expenditure on exercise days increased by 443 ± 126 kcal/d and 239 ± 152 kcal/d for aerobic and resistance exercise, respectively (p < 0.01). Non-exercise moderate-to-vigorous PA, however, decreased on aerobic exercise days (−148 ± 161 kcal/d; p = 0.03). There was no change in total daily energy expenditure and PA on non-exercise days with aerobic exercise while resistance exercise was associated with an increase in moderate-to-vigorous PA during non-exercise days (216 ± 178 kcal/d, p = 0.01). Results of the present study suggest a compensatory reduction in PA in response to aerobic exercise. Resistance exercise, on the other hand, appears to facilitate non-exercise PA, particularly on non-exercise days, which may lead to more sustainable adaptations in response to an exercise program.

## Background

The benefits of physical activity (PA) regarding cardiovascular and metabolic disease risk as well as overall quality of life have been well documented (O’Donovan et al. 2010). Insufficient PA has been associated with 1.5–3 % of direct health care costs (Oldridge 2008) and the World Health Organization considers insufficient PA as the 4th leading risk factor for global mortality (World Health Organization 2010). Nevertheless, only 26 % of US adults between 18 and 44 years of age meet current PA guidelines for aerobic and muscle strengthening activities (National Center for Health Statistics 2015). Given the decline in physical demands in daily living (e.g. occupational PA, household PA) (Archer et al. 2013; Church et al. 2011), exercise becomes an increasingly important component of an active lifestyle, particularly in young adults (Westerterp 2003). Exercise energy expenditure, however, does not simply add on to the total daily energy expenditure (TDEE) accumulated prior to an exercise intervention. Various studies have shown a smaller change in TDEE than what would have been expected based on the energy costs of specific exercise programs (Drenowatz 2015). Given the generally short duration of an exercise session, the effect of exercise on other aspects contributing to TDEE, such as non-exercise PA, need to be considered when evaluating the benefits of an exercise program. Non-exercise PA reflects activities of daily living (f.ex. locomotor activities for transportation, household chores,…) as well as unintentional activities such as fidgeting. Even in previously sedentary subjects, non-exercise activity energy expenditure was larger than the exercise induced energy expenditure (Westerterp and Plasqui 2004) and alterations in non-exercise PA have been shown to be a crucial determinant for successful weight loss in response to an exercise intervention (Manthou et al. 2010). In addition, many adults participating in exercise interventions are not able to maintain their exercise habits beyond a structured exercise intervention period and the effect of exercise on non-exercise PA could have important implications for the sustainability of exercise induced changes related to chronic disease risk.

The majority of exercise-based intervention studies relied on aerobic exercise, which may be due to the higher energy expenditure during aerobic exercise compared to resistance exercise (Strasser and Schobersberger 2011). Aerobic exercise, however, has been associated with a decline in non-exercise PA, which minimizes the exercise-induced increase in TDEE (Schutz et al. 2014; Colley et al. 2010; Rosenkilde et al. 2012). The reduction in non-exercise PA may be attributed to exercise-induced fatigue, which leads a person to be inactive during times when they would usually engage in some type of PA. In addition, participants in an exercise program may feel they can afford to rest more during the day while they are engaging in a structured exercise program. Accordingly, fatigue has been described as an emotion with the sensation of fatigue depending on current PA levels and the capacity to engage in various activities at any given time (Noakes et al. 2004). An increase in physical fitness, in response to exercise, therefore, may positively affect PA during non-exercise time. Resistance exercise, for example, has been associated with an increase in functional capacity and increased non-exercise PA (Hunter et al. 2000; Levinger et al. 2007). These studies, however, looked at elderly or participants at increased risk for chronic disease and there remains limited comparative research on the effects of resistance exercise on non-exercise PA in young adults. Even for aerobic exercise interventions research on the extent to which structured exercise leads to compensatory change in non-exercise energy expenditure remains limited resulting in a lack of research on differential effects of aerobic and resistance exercise on non-exercise PA. The purpose of this pilot study, therefore, was to examine acute and chronic effects of aerobic and resistance exercise on TDEE and non-exercise PA in young, previously sedentary men.

## Results

The sample consisted of 5 White and 4 Asian men. There was no difference in age, anthropometric characteristics and VO_2_peak by ethnicity. Four participants started with the aerobic exercise program followed by the resistance exercise program, while 5 participants completed the exercise programs in the opposite order. Baseline characteristics did not differ between participants starting with aerobic or resistance exercise. Participants completed 99.1 ± 1.6 and 98.5 ± 2.0 % of the aerobic and resistance exercise sessions, respectively. Average weartime of the SenseWear Mini Armband was 23.0 ± 0.7 h/day for 1 week during the respective measurement periods.

Table 1 shows descriptive characteristics at baseline and after the completion of both exercise programs. Based on BMI only one participant was normal weight (BMI < 25), 7 were overweight (25 ≤ BMI < 30) and 1 was obese (BMI ≥ 30). Body weight and body composition did not change significantly in response to either aerobic or resistance exercise; resulting in no change throughout the 9 months observation period. On an individual level weight change ranged between a loss of 2.6 kg and a weight gain of 3.6 kg. Change in percent body fat ranged from a loss of 2.7 % to a gain of 1.7 %. Participants did experience a significant increase in VO_2_peak (p < 0.01). Average VO_2_peak increased with the aerobic and resistance exercise program but the increase was significant only with aerobic exercise (3.2 ± 1.4 ml/kg/min; p < 0.01).

**Table 1:** Sample characteristics prior to starting any exercise and after the completion of both exercise programs

	Baseline	Follow-up	p value*
Height (cm)	176.9 ± 7.7	177.0 ± 7.8	0.476
Weight (kg)	87.4 ± 12.4	87.9 ± 14.3	0.563
BMI (kg/m^2^)	27.8 ± 2.2	27.9 ± 2.7	0.726
Fat mass (kg)	27.2 ± 7.4	27.1 ± 9.3	0.896
Lean mass (kg)	58.2 ± 7.2	58.1 ± 6.5	0.824
% Body fat	30.6 ± 4.5	30.1 ± 5.4	0.294
VO_2_ peak (ml/kg/min)	35.7 ± 5.5	40.0 ± 5.5	0.001

Values are Mean ± SD

* Dependent t-test comparing baseline values to follow-up after completion of both exercise programs

Average TDEE prior to any intervention was 3061 ± 371 kcal/day with no difference in TDEE between participants starting with resistance or aerobic exercise. There was no difference in TDEE between weekdays and the weekend at baseline (Table 2). Either exercise intervention (aerobic or resistance) resulted in a significant increase in TDEE on exercise days (p < 0.01). The average contribution of aerobic and resistance exercise to TDEE was 16.0 ± 3.1 and 11.7 ± 2.9 %, respectively. The larger exercise energy expenditure with aerobic exercise resulted in a higher TDEE during aerobic exercise days compared to resistance exercise days (p = 0.01); when exercise energy expenditure was excluded TDEE did not differ between aerobic and resistance exercise days. Resistance exercise did not have an acute effect on TDEE during non-exercise days but TDEE increased during the weekend with aerobic exercise from baseline to week 1 (p = 0.026).

**Table 2:** Total daily energy expenditure (TDEE) and exercise energy expenditure prior to and during the last week of aerobic and resistance exercise

	Aerobic exercise			Resistance exercise		
	Baseline	Week 1	Week 16	Baseline	Week 1	Week 16
TDEE EX days (kcal/day)^a,b^	3070.0 ± 439.2	3661.5 ± 426.6	3511.7 ± 497.0	3059.2 ± 427.3	3405.3 ± 512.0	3296.2 ± 396.8
Exercise EE (kcal/day)^a,b^	N/A	558.5 ± 99.0	565.9 ± 123.2	N/A	417.3 ± 129.5	366.1 ± 77.8
TDEE non EX weekdays (kcal/day) ^1^	3070.0 ± 439.2	3238.3 ± 419.1	3131.3 ± 404.5	3059.2 ± 427.3	2961.7 ± 444.1	3182.6 ± 410.2
TDEE weekend (kcal/day)	2996.8 ± 431.3	3176.1 ± 459.6	3126.8 ± 611.2	2874.5 ± 498.4	3009.5 ± 489.2	3158.1 ± 395.0

Values are Mean ± SD

*EX* supervised exercise, *EE* energy expenditure

^a^sig. difference between aerobic and resistance exercise during week 1 (p ≤ 0.01)

^b^sig. difference between aerobic and resistance exercise during week 16 (p ≤ 0.01)

**Fig. 1:**
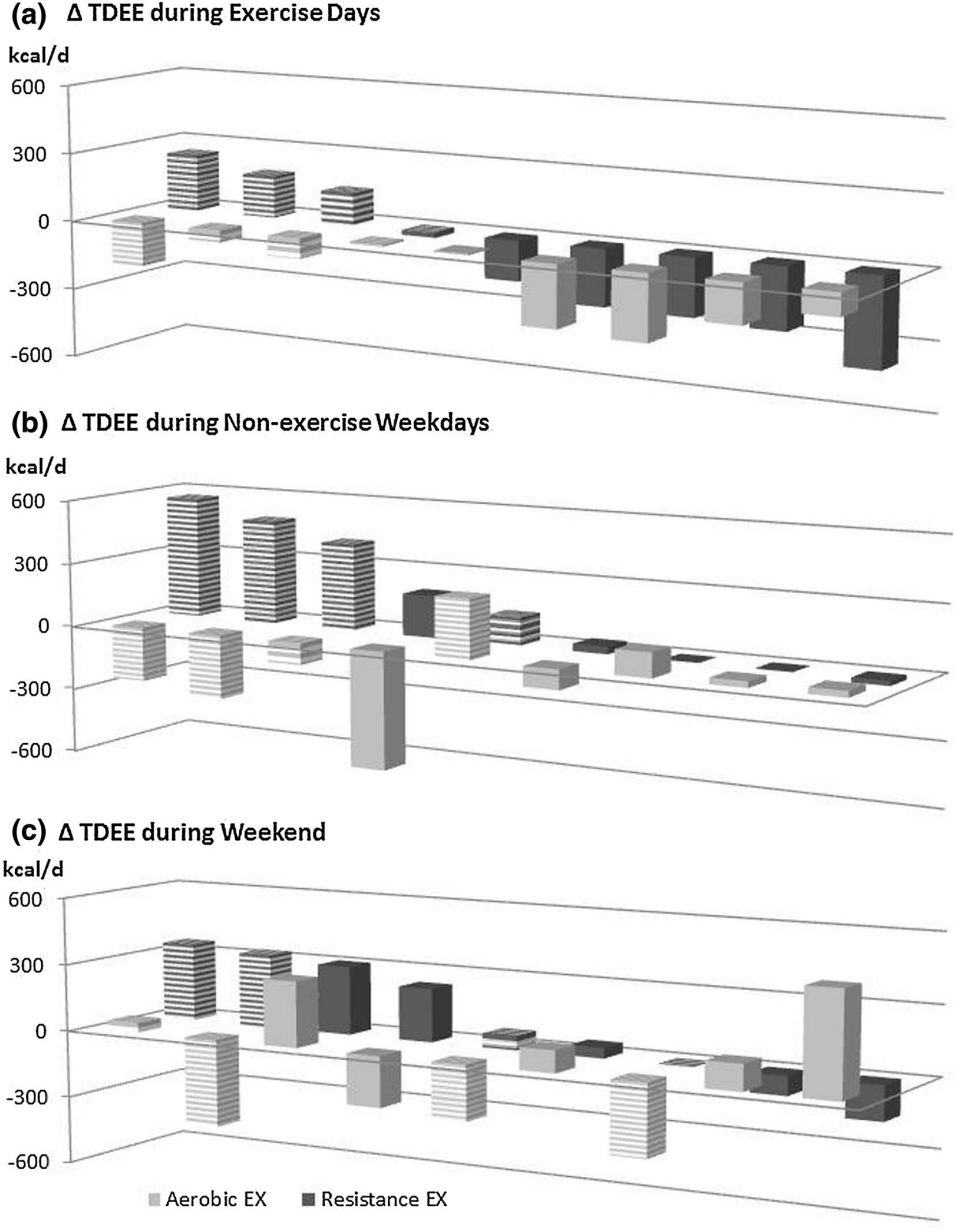
Individual change in TDEE based on linear mixed models over 16-weeks of aerobic and resistance exercise training during exercise days (**a**), non-exercise weekdays (**b**) and weekend (**c**). *Hatched bars* starting with aerobic exercise, *solid bars* starting with resistance exercise

Linear mixed models showed a significant decline in TDEE over the 16-week exercise period with aerobic exercise on exercise days (∆_TDEE AEX_ = −126.6 ± 109.2 kcal/d; p < 0.01), even though exercise energy expenditure did not change significantly. No significant change occurred in TDEE on exercise days with resistance exercise. On non-exercise weekdays TDEE increased with resistance exercise (∆_TDEE REX_ = 199.1 ± 229.1 kcal/d; p = 0.03), while there was no significant change in TDEE on non-exercise days with aerobic exercise. There was, however, a large individual variability in change in TDEE during exercise and non-exercise days in response to either exercise intervention (Fig. 1). The order of exercise engagement did not affect the response to aerobic exercise. Participants starting with aerobic exercise, however, showed an increase in TDEE over time with resistance exercise, which may be attributed to higher fitness levels. Exercise order, however, did not affect the progression of TDEE during weekends.

**Fig. 2:**
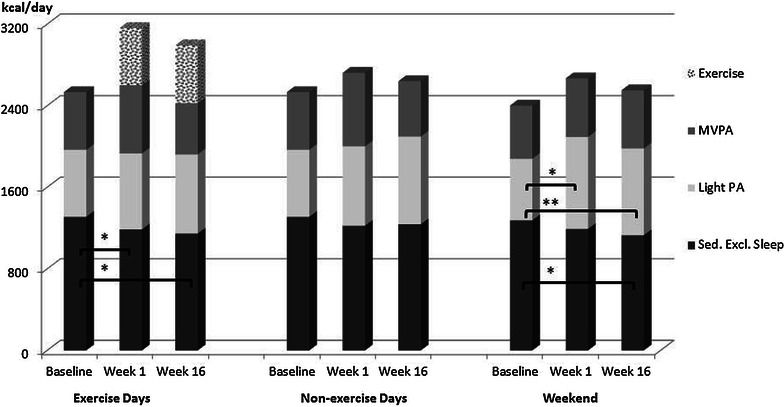
Energy expenditure at different intensities at baseline, week 1 and week 16 with aerobic exercise separately for exercise days, non-exercise weekdays and the weekend. *MVPA* moderate-to-vigorous intensity physical activity, *Sed Excl Sleep* sedentary energy expenditure excluding sleep. *p < 0.05, **p < 0.01

**Fig. 3:**
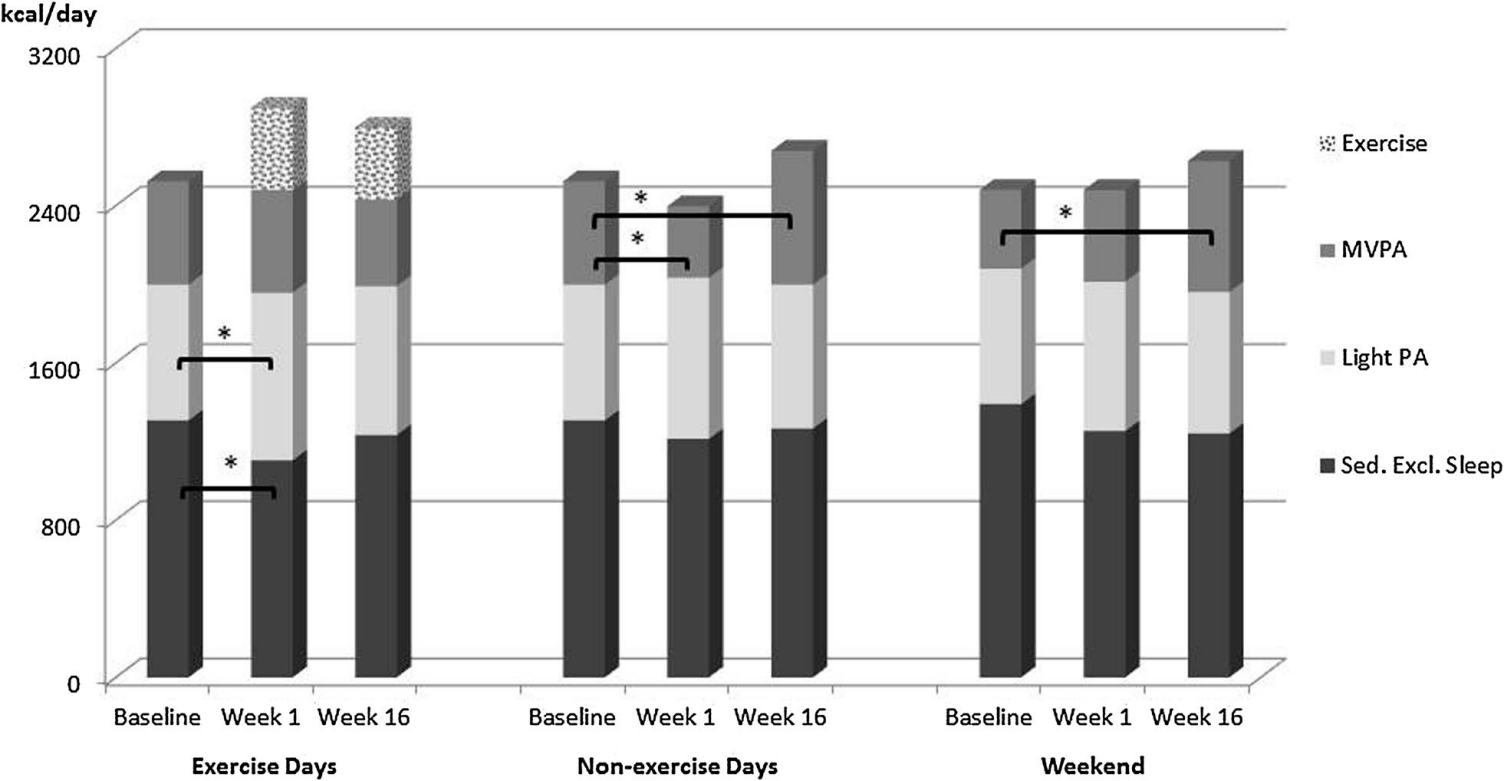
Energy expenditure at different intensities at baseline, week 1 and week 16 with resistance exercise separately for exercise days, non-exercise weekdays and the weekend. *MVPA* moderate-to-vigorous intensity physical activity, *Sed Excl Sleep* sedentary energy expenditure excluding sleep. *p < 0.05

The higher TDEE during exercise days in week 1 compared to baseline was due to the added energy expenditure of the exercise bout, which also resulted in an increased energy expenditure in moderate-to-vigorous intensity PA (∆_MVPA AEX_ = 664.2 ± 127.8, ∆_MVPA REX_ = 413.6 ± 243.2; p < 0.01) (Figs. 2, 3). When exercise energy expenditure was excluded, energy expenditure in MVPA did not differ between exercise and non-exercise days. Energy expenditure during sedentary pursuits decreased on exercise days with either exercise regimen from baseline to week 1 (∆EE_Sed AEX_ = −123.2 ± 159.5 kcal/d, ∆EE_Sed REX_ = −202.9.2 ± 194.0 kcal/d; p < 0.05). Further, there was an increase in energy expenditure in light PA on exercise days with resistance exercise (∆EE_light REX_ = 143.9 ± 168.5 kcal/d; p = 0.03). MVPA on non-exercise days, on the other hand, decreased from baseline to week 1 with resistance exercise (∆EE_MVPA REX_ = −162.4 ± 204.3 kcal/d; p = 0.04) while aerobic exercise was associated with an increase in light PA during the weekend from baseline to week 1 (∆EE_light AEX_ = 298.1 ± 202.8 kcal/d; p < 0.01) and no change in MVPA.

Throughout the 16-week exercise period, energy expenditure in light PA and during sedentary pursuits did not change significantly. MVPA, however, decreased significantly from week 1 to week 16 on exercise days with aerobic exercise (∆EE_MVPA AEX_ = −140.0 ± 174.9 kcal/d; p = 0.04), despite a lack of change in exercise energy expenditure (Fig. 2). Nevertheless, MVPA, including exercise, remained higher on exercise days compared to non-exercise days. No change was observed in MVPA on exercise days with resistance exercise (Fig. 3). During non-exercise days (weekdays and weekend) there was an increase in energy expenditure in MVPA with resistance exercise (Weekday ∆EE_MVPA REX_ = 270.9 ± 200.8 kcal/d, Weekend ∆EE_MVPA REX_ = 160.8 ± 154.3 kcal/d; p ≤ 0.01). After exercise energy expenditure was excluded, energy expenditure in MVPA on non-exercise days was higher than on exercise days in week 16 (p ≤ 0.01).

## Discussion

Evidence on the effects of exercise on non-exercise PA remains inconclusive (Drenowatz 2015). Inconsistent results may in part be due to differences in the study population but could also be attributed to differences in exercise regimen. The present study shows several interesting results regarding the association of two different exercise regimens with TDEE and PA. As has been shown previously, exercise energy expenditure is higher during aerobic exercise compared to resistance exercise (Strasser and Schobersberger 2011), resulting in a higher TDEE with aerobic exercise. Results of the present study further indicate that aerobic exercise stimulates an increase in PA during non-exercise days at the beginning of an exercise intervention. Resistance exercise, on the other hand, was associated with a reduction in PA in the short-term. In the long-term, resistance exercise, however, appears to stimulate higher PA levels during non-exercise days while no such effect was observed with aerobic exercise. In fact, there may be a compensatory reduction in non-exercise PA on exercise days with aerobic exercise, which was not observed with resistance exercise. Previous studies, particularly those with prolonged exercise engagement, did not show any compensatory adaptations in response to aerobic exercise (Hollowell et al. 2009; Rangan et al. 2011; Turner et al. 2010; Willis et al. 2014). These studies, however, did not differentiate between exercise and non-exercise days, which does not provide a complete picture of behavioral adaptations in response to an exercise intervention. The use of average values over an entire week in the present study would not have shown any significant changes in TDEE or PA from baseline to 16-week follow-up either after exercise energy expenditure was excluded (results not shown). Considering exercise and non-exercise days separately shows a more diverse pattern. In addition, the present study shows a difference in the timing of compensatory adaptations in response to aerobic and resistance exercise.

A single exercise bout was generally not associated with a change in non-exercise PA (Alahmadi et al. 2011; Cadieux et al. 2014; Sim et al. 2013), resulting in an increase in TDEE. Alahmadi et al. (2011), however, showed an increase in non-exercise PA 2 days after an aerobic exercise bout, which is comparable to the increase in light PA observed during the weekend after the first week of aerobic exercise. The increase in PA may be due to feeling more energetic after starting an exercise intervention (Church et al. 2007; Westerterp 2008). This may stimulate an increase in PA outside the exercise intervention, particularly on the weekend, when participants may have more freedom to choose between active or sedentary pursuits. Further, it has been argued that exercise positively affects mood and participants may increase their PA levels on non-exercise days to experience a similar psychological lift (Ekkekakis et al. 2005). From a physiological perspective exercise has been associated with a stimulation of the sympathetic nervous system, which may contribute to an increase in TDEE in the short-term. A prolonged engagement in similar exercise, however, may lead to adaptive reductions in sympathetic activity, resulting in lower resting energy expenditure (Johannsen et al. 2012) and TDEE (Thompson and Blanton 1987). These results, however, only looked at aerobic exercise and the present study indicates a different response with resistance exercise. The initial decline in non-exercise PA with resistance exercise may be attributed to an increased feeling of discomfort due to delayed muscular soreness, which can persist for several days after starting a new exercise routine (Cheung et al. 2003). This sensation of discomfort may override the previously described beneficial aspects that have been shown with aerobic exercise.

In the long-term resistance exercise, however, was associated with an increase in non-exercise PA, which has been attributed to an increase in functional capacity (Vincent et al. 2002; Hunter et al. 2000; Braith and Stewart 2006). The intermittent nature of resistance exercise may also more accurately resemble activities of daily living, which could lead to positive effects on non-exercise PA beyond those attributed to increased muscular strength. In addition, the lower energy expenditure during resistance exercise may induce less fatigue on exercise days, requiring less time to recover, once participants are accustomed to the exercise routine. It has further been argued that compensatory adaptations in non-exercise PA only occur when the disruption in energy balance exceeds a certain threshold (Rosenkilde et al. 2012), and the exercise energy expenditure during resistance exercise may be insufficient to achieve this threshold. Resistance exercise has also been associated with a higher energy intake following a single exercise bout compared to aerobic exercise (Cadieux et al. 2014), which would further minimize the energy gap (i.e. the difference between energy expenditure and energy intake) and reduce the stimulus for a reduction in non-exercise energy expenditure in order to maintain energy balance. Aerobic exercise, particularly of higher intensity, on the other hand, has been associated with a reduction in appetite, which has been attributed to a delay in gastric emptying (Westerterp 1998; Horner et al. 2015). A higher energy expenditure with aerobic exercise along with a potentially decreased energy intake following the exercise session results in a larger disruption of energy balance and, therefore, may be more likely to stimulate compensatory adaptations in non-exercise PA on aerobic exercise days to maintain energy balance. The desire of the human body to maintain energy balance was also indicated by results of the present study as participants did not experience a significant change in body weight and body composition throughout the entire study period despite an increase in TDEE.

Results of the present study also indicate that higher cardiorespiratory fitness is associated with smaller compensatory adaptations in response to exercise. This has been attributed to a higher exercise tolerance, which would mitigate the negative effects of exercise on non-exercise PA (Donnelly and Smith 2005). Less discomfort during the exercise and faster recovery post-exercise may further contribute to higher non-exercise PA. Higher fitness levels are also associated with a better coupling of energy intake and energy expenditure (Cook and Schoeller 2011), which facilitates maintenance of energy balance. Further, there is evidence for an increase in energy intake in response to exercise interventions, which has been used to explain the smaller than anticipated weight loss observed in most exercise interventions (Blundell et al. 2015; Hopkins et al. 2014). A smaller energy gap in response to an exercise intervention, however, could allow for less pronounced changes in non-exercise PA. Unfortunately, the small sample size of the present study did not allow to statistically test for differences in compensatory adaptations by fitness level or by improvement in fitness. Similarly, it was not possible to examine participants separately by change in energy balance or change in body composition. While the small sample size certainly limits generalizability of the results, the strong adherence to the exercise protocol and the fact that all exercise sessions were supervised is a considerable strength of the study. In addition, compliance with the measurement protocol was high as indicated by weartime of the activity monitors. Using multiple time points also allowed for an evaluation of change in energy expenditure and PA throughout a 16-week exercise period, rather than only comparing pre- and post-intervention measurements. Further, the differentiation between exercise and non-exercise days provided new insights into the association between exercise and non-exercise PA in previously sedentary adults. Future studies, however, should track motivation to exercise and exercise intensity more closely throughout the intervention period, as participants tended to work at the lower end of their respective exercise intensity towards the end of the intervention, particularly during resistance exercise. This problem may also be addressed by including strength related measures, such as an assessment of grip strength and 1-repetition maximum for the various exercises in addition to the assessment of aerobic capacity.

## Conclusions

Given the lack of research on differential effects of aerobic and resistance exercise, this study provides valuable information on compensatory adaptations in non-exercise PA, which should be considered in the development of exercise-based intervention programs. Even though aerobic exercise results in a higher energy expenditure during the exercise bout compared to resistance exercise, results of the present study emphasize the benefits of resistance exercise in the long-term, due to the beneficial effect on non-exercise PA. Resistance exercise has also been associated with a more pronounced increase in resting metabolic rate compared to aerobic exercise (Greer et al. 2015), which further contributes to a higher TDEE after the completion of a resistance exercise program. It should, however, be considered that energy balance was maintained in the present study and results may differ when participants experience a negative energy balance with a change in body composition. The present study suggests a stimulation of non-exercise PA with aerobic exercise in the short-term while in the long-term greater benefits may occur with resistance exercise. Accordingly, Rangan et al. (2011) showed the largest increase in non-exercise PA with a combination of aerobic and resistance exercise. Given that non-exercise PA is an important contributor to individual variability in change in weight and cardiorespiratory fitness (Hautala et al. 2012; Manthou et al. 2010) it is crucial to conduct further research on the effects of different exercise regimen on non-exercise PA to enhance the understanding of the role of exercise in the regulation of energy balance.

## Methods

Nine previously sedentary men (27.0 ± 3.3 years) completed a 16-week aerobic exercise program and a 16-week resistance exercise program in random order. Exercise programs were separated by a minimum of 6 weeks with participants not engaging in any structured exercise. Participants were recruited via e-mail listservs and word of mouth. In order to be eligible for the study, participants needed to be sedentary and free of any major chronic diseases. Sedentariness was defined as participating in less than 60 min of structured exercise per week (Jakicic et al. 2011). In addition participants needed to be weight stable (<3 % change in body weight during the previous 3 months) and not take any medications that are known to affect body composition or blood pressure (i.e. beta-blocker, Synthroid). The study protocol was approved by the University’s Institutional Review Board and all participants provided written informed consent.

Each exercise program consisted of 3 supervised exercise sessions per week that were completed between Monday and Friday. Prior to the beginning of each exercise program participants completed one orientation exercise session to introduce them to the exercise equipment and determine the appropriate exercise intensity. Aerobic exercise sessions were performed on a treadmill (True Fitness Technology, St. Louis, MO) and lasted 60 min, including a 10-min warm-up and 5-min cool-down. The warm-up consisted of walking at 3.5 mph at 0 % incline. Following the warm-up, participants exercised for 45 min at an intensity between 60 and 75 % of their maximum heart rate, which was determined via a graded exercise test. Heart rate was recorded continuously throughout the entire exercise session via heart rate telemetry (Polar RS 400, Polar Electro Inc., Lake Success, NY). Every 5–10 min laboratory staff verified that participants were exercising at the appropriate intensity and treadmill speed and incline were adjusted if necessary. The cool-down consisted of walking at a self-selected pace at 0 % incline. Resistance exercise sessions consisted of the same warm-up and cool down as described for aerobic exercise. The resistance exercise routine consisted of 10 machine-based exercises targeting the whole body. Specifically, participants completed 3 sets with 8–12 repetitions of 5 upper body exercises (bench press, lat pull, shoulder press, biceps curl, triceps extension), 3 lower body exercises (leg press, leg extension, leg curl) and 2 core exercises (abdominal crunches, back extension). Resistance for individual exercises was increased when participants completed 3 sets of 12 repetitions on 2 consecutive exercise days in order to adjust for adaptations in response to the training. Total exercise time for resistance exercise sessions, including warm-up and cool-down, was 67 ± 12 min. As for aerobic exercise, heart rate was recorded for every exercise session.

TDEE and energy expenditure at various intensities were measured with the SenseWear Mini Armband (SWA, BodyMedia Inc., Pittsburgh, PA, USA), which was worn for a minimum of 8 days prior to starting each exercise program as well as during week 1, week 8 and week 16 of each exercise program. The SWA measures tri-axial accelerometry, galvanic skin response, heat flux, skin temperature and near body temperature to estimate minute-by-minute energy expenditure. Validation studies, using indirect calorimetry or doubly labelled as criterion measures have shown that the SWA provides accurate estimations of energy expenditure in healthy adults (Johannsen et al. 2010; St-Onge et al. 2007; Casiraghi et al. 2013). Participants were asked to wear the SWA for 24 h/day and only remove it during periods when it might get wet, such as taking a shower. Compliance was defined as 7 days (incl. Saturday and Sunday) with more than 18 h of weartime. SenseWear’s proprietary algorithm (version 8.0 professional) was used to determine TDEE (kcal/d) as well as energy expenditure during sedentary pursuits, excluding sleep (sedentary < 1.5 METs), during light PA (1.5 METs ≤ light PA < 3 METs), moderate PA (3 METs ≤ MPA < 6 METs), and vigorous PA (VPA ≥ 6 METs).

Height (cm) and body weight (kg) were measured after a 12-h fast with the participants wearing surgical scrubs and in bare feet at baseline (prior to the start of each exercise program), during week 8 and at the end of each exercise program. Measurements were performed in duplicates to the nearest 0.1 cm and 0.1 kg, respectively with average values used to calculate BMI (kg/m^2^). In addition, body weight was measured prior to every exercise session with participants wearing workout clothes and in bare feet. Fat mass and lean mass were measured prior to and at the end of each exercise program via dual energy x-ray absorptiometry (DXA; GE Healthcare Lunar model 8743, Waukesha, WI). Subsequently, percent body fat was calculated (fat mass/body weight).

A treadmill-based graded exercise test to maximal exertion was performed prior to and upon completion of each exercise program using a modified Bruce Protocol. The protocol consisted of 2 min stages starting with a speed of 1.7 mph at 0 % incline. For the next 2 stages speed remained at 1.7 mph while the incline increased to 5 and 10 %, respectively. For the remaining stages speed was set at 2.5, 3.4, 4.2, 5.0, 5.5 and 6.0 mph with incline increasing by 2 % at every stage. Participants were asked to continue until volitional exhaustion. Oxygen consumption was monitored continuously via indirect calorimetry (True One 2400, Parvo Medics, Sandy, UT) and VO_2_peak was determined as the highest value observed during the test when at least two of the following criteria were met: rate of perceived exertion ≥17, maximum achieved heart rate >90 % bpm of age predicted heart rate, respiratory quotient ≥1.15, plateau in heart rate and plateau in oxygen consumption with increasing workload.

### Statistical analysis

Change in body composition as well as VO2peak was examined via repeated measures analyses separately for each exercise intervention and over the entire observation period, adjusting for the order of exercise participation. Dependent t-tests were used to examine the acute effect (baseline to week 1) of aerobic and resistance exercise on TDEE and energy expenditure at different intensities, separately for exercise days, non-exercise weekdays and weekend days. Long-term effects over each 16-week exercise period were examined via Linear Mixed Models, adjusting for change in body weight. All statistical analyses were performed with IBM SPSS Statistics for Windows (version 22.0; IBM Corp., Armonk, NY, USA) using a *p* value of 0.05 for statistical significance.

## Abbreviations

MVPA: moderate-to-vigorous physical activity
PA: physical activity
SWA: sensewear mini armband
TDEE: total daily energy expenditure
